# Advances in the allostery of angiotensin II type 1 receptor

**DOI:** 10.1186/s13578-023-01063-x

**Published:** 2023-06-17

**Authors:** Xi Zhang, Suli Zhang, Meili Wang, Hao Chen, Huirong Liu

**Affiliations:** 1 Department of Physiology & Pathophysiology, School of Basic Medical Sciences, Capital Medical University, Beijing, 100069 People’s Republic of China; 2 Beijing Key Laboratory of Metabolic Disorders Related Cardiovascular Disease, Capital Medical University, Beijing, 100069 People’s Republic of China; 3 Department of Physiology & Pathophysiology, School of Basic Medical Sciences, Capital Medical University, 10 Xitoutiao, You An Men Street, Beijing, 100069 China

**Keywords:** Angiotensin II type 1 receptor, Conformation, Allostery, Biased ligands, Dimers, Allosteric pocket

## Abstract

Angiotensin II type 1 receptor (AT1R) is a promising therapeutic target for cardiovascular diseases. Compared with orthosteric ligands, allosteric modulators attract considerable attention for drug development due to their unique advantages of high selectivity and safety. However, no allosteric modulators of AT1R have been applied in clinical trials up to now. Except for the classical allosteric modulators of AT1R such as antibody, peptides and amino acids, cholesterol and biased allosteric modulators, there are non-classical allosteric modes including the ligand-independent allosteric mode, and allosteric mode of biased agonists and dimers. In addition, finding the allosteric pockets based on AT1R conformational change and interaction interface of dimers are the future of drug design. In this review, we summarize the different allosteric mode of AT1R, with a view to contribute to the development and utilization of drugs targeting AT1R allostery.

## Introduction

G protein-coupled receptors (GPCRs) are the largest family of membrane receptors with seven transmembrane (TM) α-helices. GPCR-tageting Drugs account for approximately 34% of all FDA-approved drugs [1]. Angiotensin II type 1 receptor (AT1R) is a member of the rhodopsin family of GPCRs. It possesses an extracellular N terminus, three extra- and intracellular loops (ECL1-3 and ICL1-3), an amphipathic helix 8 (H8), and an intracellular C terminus. Abnormal activation of AT1R leads to various diseases, such as hypertension, coronary artery disease, arrhythmia, and diabetic nephropathy [2, 3]. Therefore, AT1R is becoming a target for drug development. However, functional diversity exists due to the signaling diversity of AT1R, which poses a challenge for the application in diseases.

As an important mechanism of the signal diversity, allosteric modulation occurs when a variety of extracellular stimuli (ions, lipids, peptides, proteins, autoantibodies, etc.) interact with allosteric sites of GPCR, which affects the orthosteric ligands [4–6]. In addition to classical allostery, allosteric regulation has many other forms, such as the activation mode of biased ligands and dimers. The phenomenon that GPCRs preferentially form a certain conformation and select intracellular sensors to produce different signal activation is termed “biased agonism” or “functional selectivity”, which is a natural result of allosteric selection. In other words, biased activation implies the cytosol-directed allostery [7]. Besides, GPCR dimerization is a more subtle form of allosteric regulation, breaking the traditional concept of allosteric activators, that the initiator is a monomer in the dimer or its ligand, and the allosteric information is transmitted laterally [8–10].

As a valuable target for drug development, allosteric modulation of AT1R is still in its infancy. To better understand the mechanism behind the functional diversity of AT1R and expedite the development of novel allosteric drugs, this paper reviews the current status of AT1R allosteric regulation, focusing on the classical and non-classical allostery, and the allosteric pockets of AT1R.

## Classical allostery and classical allosteric modulators of AT1R

Classical allostery suggests that binding an allosteric modulator to an allosteric site can modulate the properties of an orthosteric ligand in the same protein (enzyme, receptor, etc.) [10]. The allosteric modulators bind to the allosteric sites and subsequently regulate the binding of the orthosteric ligands to the orthosteric sites. Classically, most GPCR drugs target the orthosteric sites. Orthosteric ligands of GPCR are divided into three categories: (1) agonists that can activate GPCR; (2) inverse agonists that inhibit the constitutive activity of GPCR; and (3) antagonists that occupy the orthosteric site without effect) [11]. Except for the orthosteric sites, the binding sites are called allosteric sites or allosteric pockets, which bind to allosteric ligands [12]. Allosteric ligands transmit allosteric signals to functional sites through atomic fluctuations, amino acid residue networks, or domain movements, thereby increasing, maintaining, or decreasing the affinity and/or efficacy of orthosteric ligands [5, 13, 14]. During biological evolution, the orthosteric sites remained highly conserved. In contrast, the allosteric sites are less conserved and have structural diversity, not driven by evolutionary pressures. Meanwhile, allosteric modulators have higher target specificity, better spatiotemporal specificity (synergism with endogenous ligands), and functional specificity, thereby providing higher selectivity and better control of receptor dynamics [15, 16]. Moreover, allosteric modulators are saturable and do not disrupt signaling networks with increasing doses. This feature reduces the risk of side effects and offers potential therapeutic advantages [17].

Allosteric modulators trigger the allosteric influence of the receptor activated by orthosteric modulators. Allosteric modulators are classified into three types based on their effects: positive allosteric modulators (PAM), negative allosteric modulators (NAM), and neutral allosteric ligands (NAL, also known as silent allosteric modulators (SAM)) [16, 18]. In the absence of orthosteric modulators, some PAM, known as ago-allosteric modulators (ago-PAM), can activate the receptor alone [19]. Biased allosteric modulators (BAM) alter the activation mode of receptors through specific signaling [20]. Here, we summarize the classical allosteric modulators of AT1R.

### Antibody

**Fig. 1:**
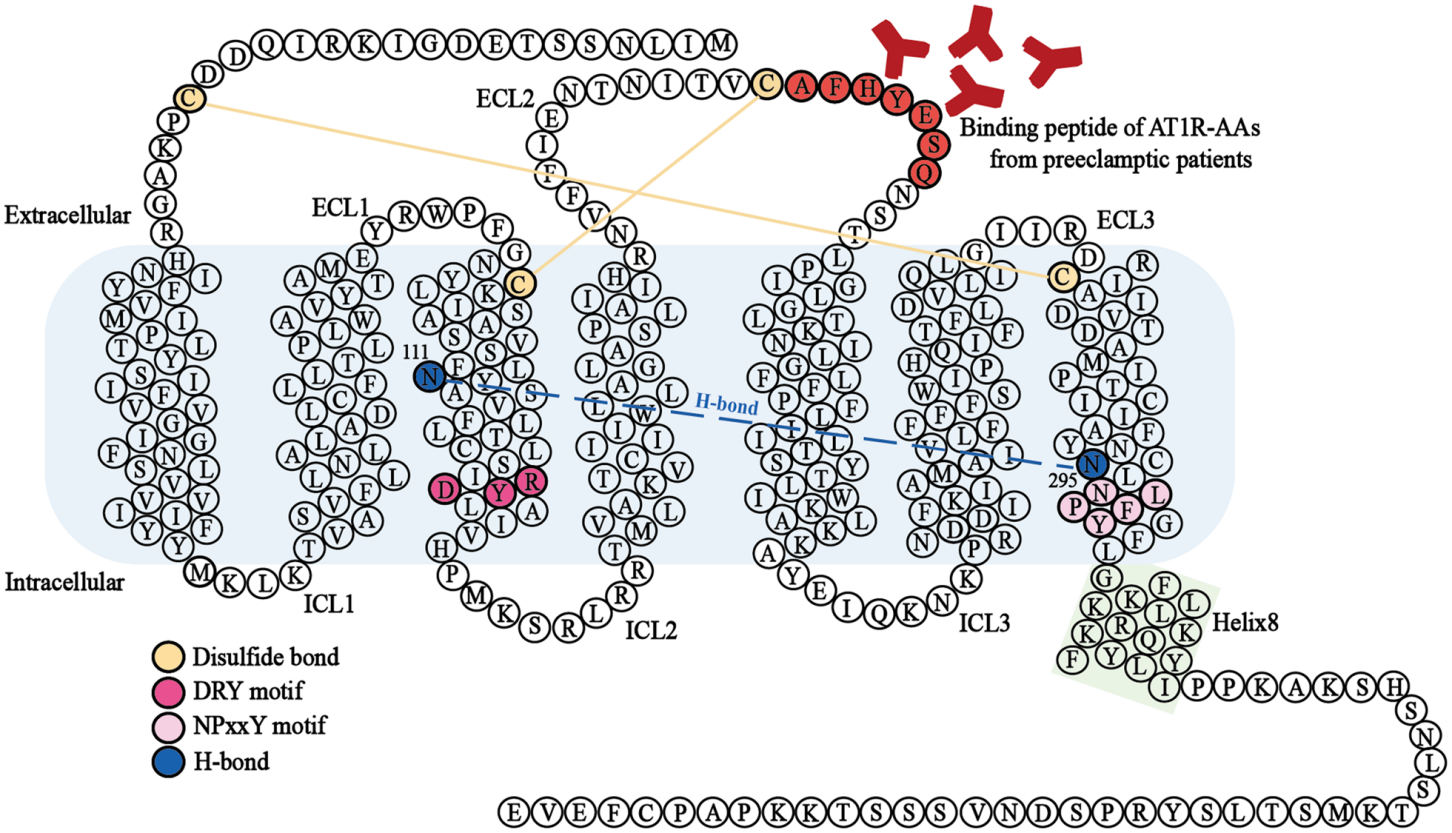
The protein structure of human AT1R. Secondary structure of human AT1R with depiction of different motifs and important sites. Cys18–Cys274, the disulfide bond, stabilizes the N terminus and ECL3. And Cys101–Cys180 stabilizes TM3 and ECL2. The DRY motif and NPxxY motif, the microswitches of AT1R, are considered to participate in receptor activation. The interaction, N111^3.35^ hydrogen bonds with N295^7.46^, stabilizes the inactive state of AT1R.

Antibodies produced against self-antigen are known as autoantibodies (AAs). The majority of AAs against GPCR (GPCR-AAs) are allosteric modulators that enhance or reduce the effect of orthosteric ligands and alter the endogenous biological properties of the receptor. They possess unique and complex pharmacological properties [21]. AT1R autoantibodies (AT1R-AAs) acted as ago-PAM and were first identified in patients with preeclampsia [22] (Fig. 1). AT1R-AAs are deemed to exert pathogenic effects and have been implicated in various diseases such as metabolic syndrome [23], malignant hypertension [24], unstable angina (UA) [25], renal transplant rejection [26], frailty [27], Alzheimer’s disease [28], and COVID-19 [29–31]. Angiotensin II (AngII), the orthosteric ligand of AT1R, activates G protein and β-arrestin signaling cascades [32, 33]. Unlike AngII, AT1R-AAs bind to the ECL2 of AT1R and independently activate the receptor for a long period of time by inhibiting β-arrestin1/2 recruitment and AT1R internalization. They can cause sustained vasoconstriction, tissue fibrosis, and migration of immune regulatory cells [34, 35]. This may be due to the fact that AT1R forms a conformation that does not easily bind to β-arrestin1/2 after being targeted by AT1R-AAs. Therefore, the effect of AT1R-AAs may be related to a type II hypersensitivity response, similar to autobodies of thyroid-stimulating hormone receptor (TSHR) that can overactivate TSHR in Graves’ disease [36].

It has been shown that AT1R-AAs amplify AngII-induced vasoconstrictor response and increase the sensitivity of AngII to AT1R, but the mechanism is unclear [37–39]. The two disulfide bonds of AT1R, Cys18-Cys274 and Cys101-Cys180, stabilize the conformation of the N terminus and ECL2 [40, 41] (Fig. 1). AT1R-AAs may affect the strain on the disulfide bond by binding to the ECL2 epitope. Cascade reactions of the receptor core alter the movement of the transmembrane helix, thereby affecting the recruitment of intracellular sensors. Alternatively, AT1R-AAs may have a microeffect on the binding residues of AngII to AT1R, thereby promoting the binding affinity and efficacy of AngII to the receptor or hindering the removal of AngII from the pocket.

Unexpectedly, AT1R-AAs were found to exert protective effects on COVID-19 possibly by interfering with the action of AngII and reducing the inflammatory response in the acute phase response of COVID-19 [42]. Uncertainty regarding the antigenic epitopes of the antibodies is a possible reason for the different effects of AT1R-AAs. It has been shown that unlike the agonistic effect of autoantibodies against ECL2, a monoclonal antibody against the N-terminal of AT1R, 6313/G2, inhibited AngII-induced cell proliferation [43]. Therefore, antibodies generated against different domains of a receptor can exert different modulating effects.

**Fig. 2:**
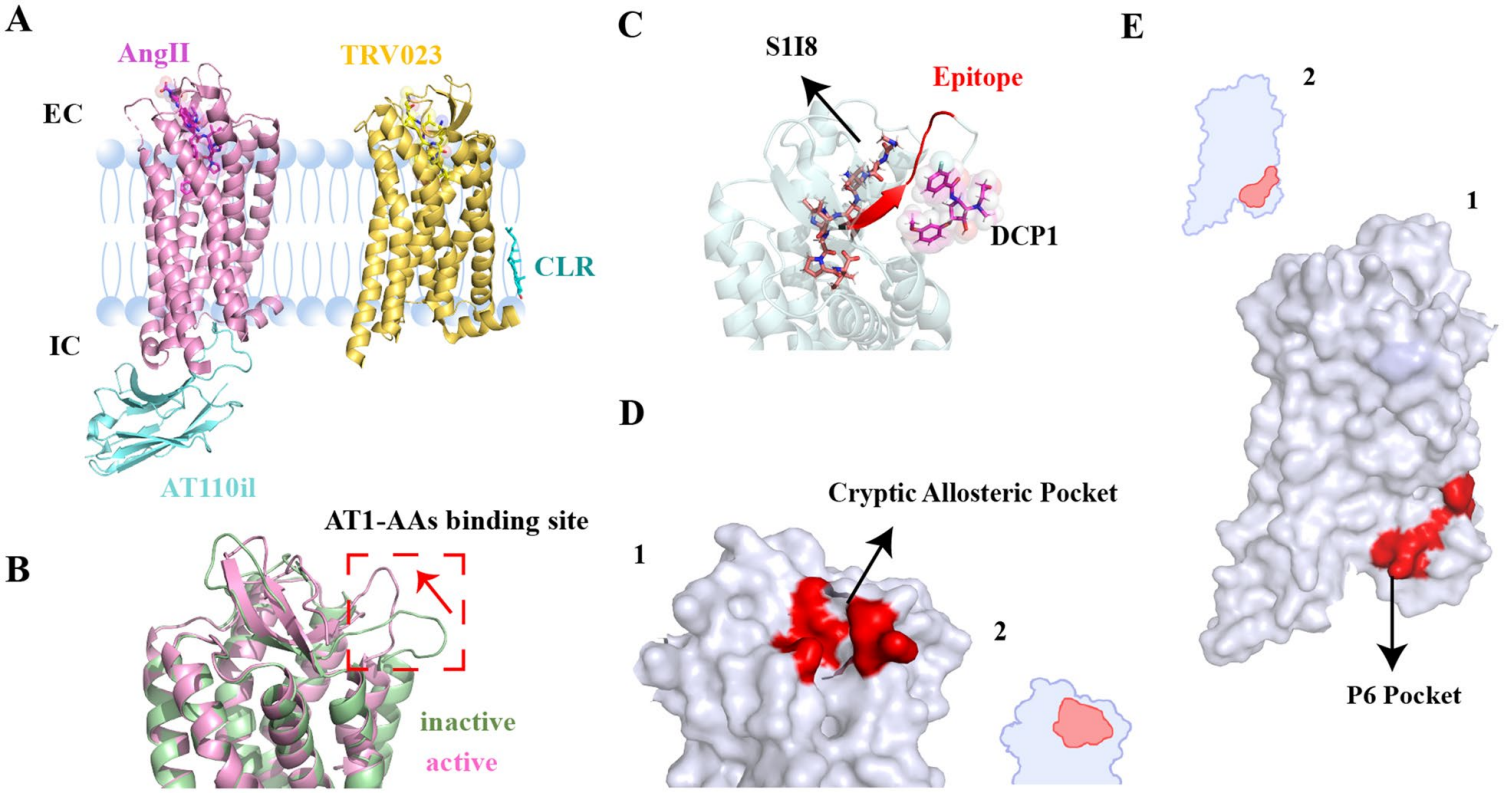
Allosteric modulators and pockets of AT1R. **A** AT110il and CLR are allosteric modulators of AT1R. **B** The active and inactive states of AT1R shows significant structural change in the binding site of AT1R-AAs. **C** A cryptic allosteric pocket is formed during MD simulation. **D**1) S1I8-bound AT1R with allosteric compound DCP1 and AT1R-AAs binding epitope are highlighted. **D**2) The cartoon picture of D1). **E**1) A potential cryptic allosteric pocket P6 is observed by using Fpocket. P6 is only identified during the movement of H8. **E**2) The cartoon picture of E1).

Researchers often use antibodies to stabilize the receptor and obtain the active crystal structure. These antibodies, always act as PAM for the receptor. To stabilize the AT1R-activated conformation, the nanobody AT110i1 was used to target ICL2 of AT1R by a synthetic yeast-displayed library. AT110i1 increased the binding affinity of the partial agonist S1I8, the full agonist AngII, and β-arrestin-biased agonists TRV026 and TRV023 to AT1R, and made the ligand-receptor complex more stable [44, 45] (Fig. 2A). Although the nanobody AT110i1 fixes the activated AT1R by simulating the binding of Gq, it is possibly differ from the activated conformation in the physiological state. Identification of the structure of the true complexity of AT1R with sensors such as GPCR kinase (GRK) and β-arrestin can help better understand receptor conformational change. Even the new conformational states can be unfolded, which is crucial for the design of allosteric modulators.

### Peptides and amino acids

The hemoglobin-derived peptides LVV-hemorphin-7 (LVV-H7) and homocysteine (Hcy) are newly identified PAM of AT1R. LVV-H7 was reported to enhance AT1R-mediated Gq and β-arrestin signaling produced by AngII [46]. Conformation changes are prerequisites for alterations in signaling and effects. Molecular docking and molecular dynamics (MD) simulations indicated that LVV-H7 targeted residues on the second and third intracellular loops of AT1R, thereby allosterically enhancing the binding affinity of AngII. In the presence of LVV-H7, AngII binds deeper in the orthosteric pocket, forming more hydrogen bonds, and hydrophobic and polar interactions. Moreover, the side chain of Arg126^3.50^ slightly orients toward TM6, which facilitates the opening of the G protein-binding groove and enhances G protein binding to AT1R [47]. Hcy is a sulfur-containing non-essential amino acid and the high serum level of Hcy is a risk factor for cardiovascular diseases. It has been shown that Hcy can activate AT1R directly via Arg167^4.64^ and Cys289^7.40^. Hcy allosterically interacts with AT1R through Cys289^7.40^, triggering a unique conformation of AT1R ICL2, which synergistically activates the receptor with AngII and aggravates vascular injury in the abdominal aortic aneurysm [48].

### Cholesterol

GPCR functions in a cell membrane environment where cholesterol (CLR), as a sterol-like type of lipid, is highly abundant and can directly bind to the receptor, thereby allosterically regulating the affinity and efficacy of the ligand as well as the spontaneous activity of the receptor. CLR may also indirectly affect GPCR and its signal transduction by altering the fluidity of the cell membrane. In the crystal structure of AT1R with the biased agonist TRV023 (6OS1), cholesterol interacts with the receptor at Phe39^1.43^, Phe44^1.48^, and Ser47^1.51^ of TM1 and H8 [15] (Fig. 2A). Six cholesterol molecules were identified in the AT1R-Gq structure(49). Cholesterol may directly or indirectly affect the binding of drugs to their receptors. Cholesterol can prevent the antagonistic effect of losartan on AT1R by preventing its access to AT1R [50]. Understanding the role of cholesterol in the allosteric activation of GPCR is an important step in the treatment of hypercholesterolemia.

### Biased allosteric modulators (BAM) of AT1R

The advent of BAM led to a new breakthrough in GPCR drug discovery. Unlike biased agonists that bind to the orthosteric site, BAM bind to the allosteric site, and exert pathway-specific effects, with the potential to selectively stimulate relevant signals and avoid side effects [20]. Cartilage oligomeric matrix protein (COMP) is an endogenous biased inhibitory ligand of AT1R that directly interacts with the N-terminal of AT1R via the structural domain of epidermal growth factor (EGF). COMP allosterically regulated receptor conformation and selectively inhibited AT1R/β-arrestin2 signaling in mice [51]. COMP can be used as a biased allosteric target for developing drugs for cardiovascular diseases in the future. BAM can help develop more effective and selective treatments.

## Non-classical allostery of AT1R

In addition to classical activation, AT1R can be activated by non-classical allosteric patterns, including ligand-independent allosteric mode, and allosteric mode of biased agonists and dimers. GPCRs can be spontaneously activated in the absence of ligands, known as constitutive activity [52, 53]. It has been found that allosteric modulation can occur through the constitutive activity of receptors because of constitutively active mutants (CAMs) and mechanical stretch [52, 54]. “Biased agonists” or “biased ligands” mainly activate one of the receptor-mediated downstream pathways, such as G protein-dependent or non-G protein-dependent β-arrestin pathway for biased activation [55–57]. The allostery of GPCR depends on the type of ligand and signaling protein. Different ligands can control sensor coupling and biased signal selection through allostery [58]. Allostery provides a mechanistic explanation for dimers. Compared to monomers, the allosteric process of dimers broadens the range of cellular signaling pathways and increases the complexity of GPCR signaling [59]. The obvious functional advantage of dimers is that they can function as allosteric machines. Changes in the spatial conformation of one receptor can allosterically regulate the function of another receptor, affecting the selectivity of the downstream signaling pathway of GPCR dimers and triggering a series of functional changes with various pharmacological properties [60, 61].

### Ligand-independent allosteric mode

CAMs and mechanical stretch allosterically regulate AT1R signaling. N111G-AT1R preferentially couples to Gq and increases IP production [62]. However, D74N, DRY/AAY, and N298A mutants of AT1R have strong interaction with β-arrestin2 [63]. CAMs may change the impact of TMs on the allosteric pathway, which provides effective tools for screening inverse agonists.

Mechanical stress can allosterically promote the constitutive activation of AT1R. In the absence of AngII and G protein activation, mechanical stress can induce GRK5/6-dependent β-arrestin-biased signaling as downstream of AT1R [64]. Mechanical stretch increases the binding affinity and efficiency of the biased agonist TRV120023 by stabilizing the specific β-arrestin-activated conformation of AT1R [54]. Leu212, Gln257, and Cys289 are key sites for activating AT1R by mechanical stretch [65]. Non-ligand regulation enhances multidimensional activation mode of AT1R. However, it is unclear whether mechanical stretch works independently or through mechanical components such as ion channels. AT1R activation due to mechanical stimuli is likely the result of a combined effect.

### Allosteric mode of biased agonists

AT1R-biased agonists are obtained by modifying AngII. Gq-biased agonists of AT1R include TRV055 and TRV056. β-arrestin-biased agonists include SII, TRV120023, TRV120027, etc. Interestingly, some of these β-arrestin-biased ligands exhibit unique beneficial properties. In 2002, Alice et al. identified the first β-arrestin-biased agonist of AT1R, [Sar1-Ile4-Ile8]-AngII (SII), where Asp1 of AngII was replaced by sarcosine, and Tyr4 and Phe8 were replaced by isoleucine [66]. In a rat model of cardiac ischemia-reperfusion, SII pretreatment reduced myocardial infarct size by 24% [67]. In isolated adult mouse cardiomyocytes, SII promoted positive inotropic and lusitropic responses via GRK6/β-arrestin2 [68]. And then the investigators developed TRV120023 and TRV120027 (KD = 12 nM) with higher affinity and efficiency. As a potential therapeutic agent for dilated cardiomyopathy (DCM), TRV120023 enhanced cardiac contractility by upregulating ventricular myosin light chain-2 phosphorylation, without mobilizing Ca^2+ ^[69]. Similarly, TRV120027 not only reduced mean arterial pressure but also enhanced cardiomyocyte contractility, increased cardiac performance, and preserved cardiac stroke volume [70], which was demonstrated in heart failure canines [71]. A study in pediatric heart failure (PHF) found that TRV027 induced a long-acting, strong positive inotropic effect without affecting heart rate, reactive oxygen species production, and adrenal aldosterone secretion in neonatal mouse [72]. These results support the therapeutic potential of biased agonists over ARB. Trevena, Inc. announced a clinical trial to study the effect of TRV027 on patients with COVID-19. Data from 30 patients showed that TRV027 was well-tolerated and decreased circulating D-dimer in 70% of patients. TRV027 was associated with 92% probability of a potential beneficial treatment effect, with the potential to improve COVID-19 progression-related biologic markers and clinical endpoints [73].

**Fig. 3:**
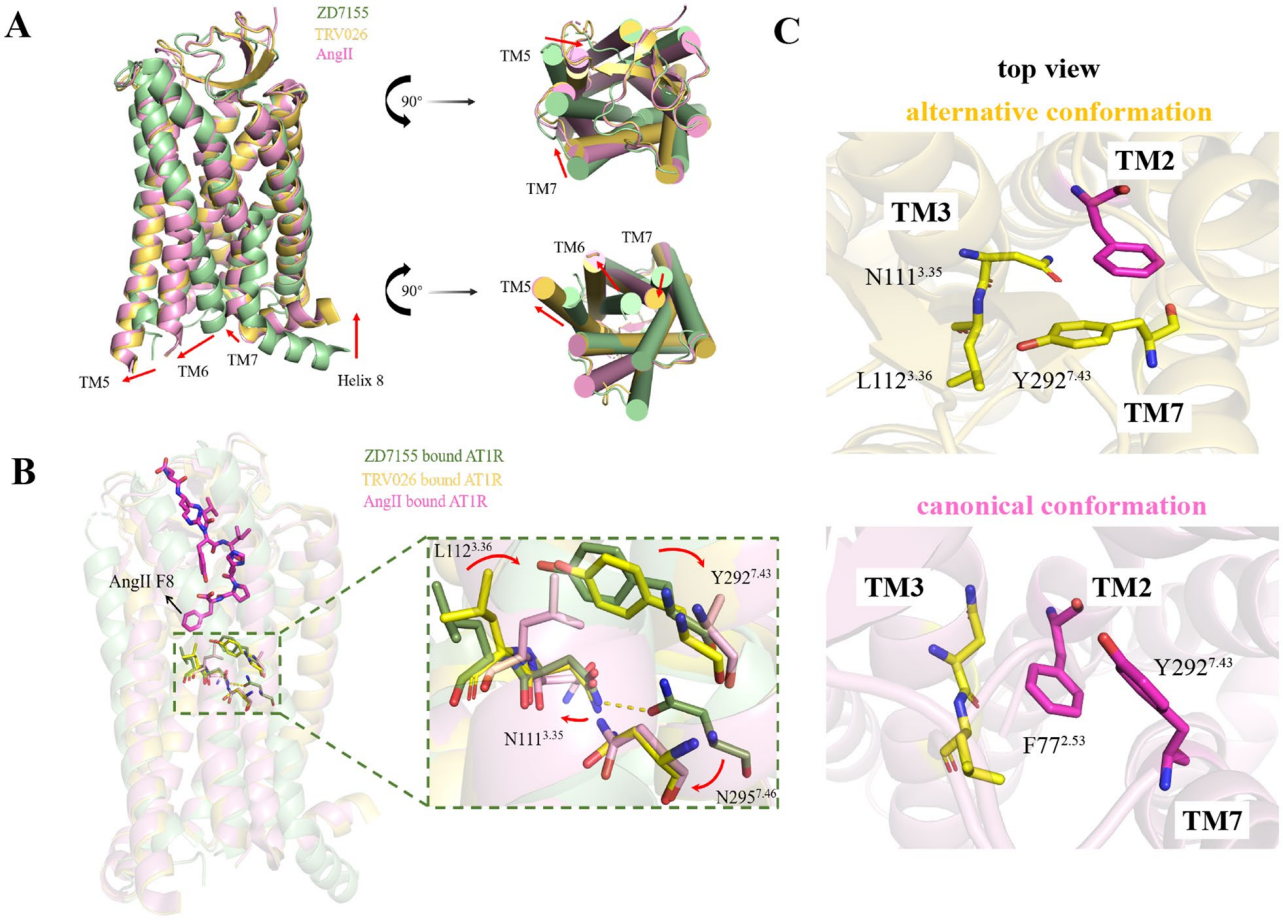
Comparison of the allosteric structures of human AT1R bound to different ligands. **A** Overall conformational changes in human AT1R with blocker ZD7155 (PDB ID: 4YAY, green), β-arrestin biased ligand TRV026 (PDB ID: 60S2, yellow) and endogenous agonist AngII (PDB ID: 6OS0, pink). In the active state, TM5 and TM6 move out of AT1R whereas TM7 moves inward of the receptor. Helix 8 adopts a position parallel to the membrane compared to the inactive conformation bent away from the membrane. Viewing from the extracellular side, TM5 and TM7 move inward of inactive AT1R. **B** Superimposed structural details of AT1R induced by different ligands. The bulky phenylalanine of AngII at position 8 pushes L112^3.36^ inward and Y292^7.43^ in a relocation. A hydrogen bond between N295^7.46^ and N111^3.35^ breaks, and the two residuces move inward. However, due to TRV026 being less deeply into the binding pocket of AT1R, it has very little effect on movement of these residuces except for N295^7.46^. **C** The alternative conformation is chosen when TM7 points toward TM3, and the canonical active conformation is chosen when TM7 points toward TM2.

However, the allosteric mechanism of the receptor for biased ligands is poorly understood. It is not clear how the ligand induces the corresponding conformation of the receptor and GPCR signal selection. The biased agonist binds to the orthosteric pocket of the receptor, activates a microswitch through allosteric pathways, triggers a conformational rearrangement of the receptor core, and initiates some intracellular sensors. Highly conserved residue motifs such as DRY and NPxxY act as microswitches. DRY motif (Asp125^3.49^-Arg126^3.50^-Tyr127^3.51^) in AT1R TM3, commonly referred to as ion lock, and NPxxY motif (Asn298^7.49^-Pro299^7.50^-Leu300^7.51^-Glu301^7.52^-Tyr302^7.53^) in TM7 are crucial for the recruitment of G-protein and receptor activation [74] (Fig. 1). Wingler et al. resolved the crystal structures of AT1R with AngII (2.9 Å), the β-arrestin-biased agonist TRV026 (2.7 Å), and TRV023 (2.8 Å) [45]. In line with previous findings, AngII adopts a vertical binding mode, reaching deep into the receptor core while contacting the extracellular surface [75]. Compared with the inactive state, in the active state, the extracellular changes of AT1R are the inward movement of TM5 and TM7, and intracellular changes of AT1R are the outward movement of TM5 and TM6, inward movement of TM7, and movement of H8 toward the cell membrane (Fig. 3A). In the AngII-AT1R structure, the phenylalanine at position 8 plays a decisive role in the initiation of allostery, triggering TM3 to rotate around the axis and Leu112^3.34^ to rotate inward to occupy position Tyr292^7.43^. Phe8 engaged Ile288^7.39^ of AT1R in van der Waals interaction, while pulling Tyr292^7.43 ^[76]. To avoid spatial limitation, Tyr292^7.43^ turns downward and the flip of Asn111^3.35^ break the hydrogen bond formed with Asn295^7.46^, which is the main constraint in the inactive state. This is believed to adapt to the insertion of Gα subunit, which is essential for Gq signaling. Tyr302^7.53^ of the NPxxY motif and Arg126^3.50^ of the DRY motif are both involved in stabilizing the activation state. However, Asn111^3.35^ is maintained near the reoriented Asn295^7.46^ in the structure of the β-arrestin-biased ligand and AT1R. It restricts TM3 and TM7 to form a blocking conformation. The binding of the β-arrestin-biased agonist triggers the TM6 transition and the conformational rearrangement of Asn295^7.46^, which is a marker of GPCR activation and may be sufficient for the conformation required for β-arrestin coupling [77] (Fig. 3B).

Based on the crystal structures, Suomivuori et al. revealed the existence of two active conformations of AT1R by MD simulations: the classical conformation and the alternative conformation. The classical conformation can bind to both Gq and β-arrestin, while the alternative conformation only binds to β-arrestin. The main difference was the orientation of TM7: the classical conformation is preferred when TM7 points to TM2; the alternative conformation is preferred when TM7 points to TM3, forming an allosteric network (Fig. 3C). In the alternative conformation, TM7 is twisted counterclockwise above the proline kink and the intracellular portion is moved toward TM3, forming a hydrogen bond between Asn46^1.50^ and Cys296^7.47^. The side chains of Tyr302^7.53^ and Arg126^3.50^ rotate downward, clashing with the α5 helix of Gq and the alternative conformation readily accommodates the β-arrestin finger loop. During the simulation, the Gq-biased agonist adopted the horizontal F8 orientation more frequently than AngII. In addition, the lack of positively charged residues in the second position prevented binding to negatively charged pocket residues Asp263^6.58^ and Asp281^7.32^ with less restriction on TM6 and greater tendency for the extracellular end of TM6 to move outward [77]. Structural allostery in AT1R-biased agonists provides a plausible explanation for their effects.

### Allosteric mode of dimers

GPCRs are known to form homodimers and heterodimers [8]. Homodimers consist of receptor subtypes of the same family. Heterodimers consist of two different types of receptors or different subtypes of the same receptor [78]. It has been shown that a negative allosteric regulation occurs between the promoters of AT1R homodimer [79]. Heterodimers, with at least two orthosteric sites and two allosteric sites, are more complex than monomers or homodimers. The formation of heterodimers provides an opportunity for their respective ligands to exert reinforcing/antagonistic effects, or even generate new signaling pathways [80].

**Fig. 4:**
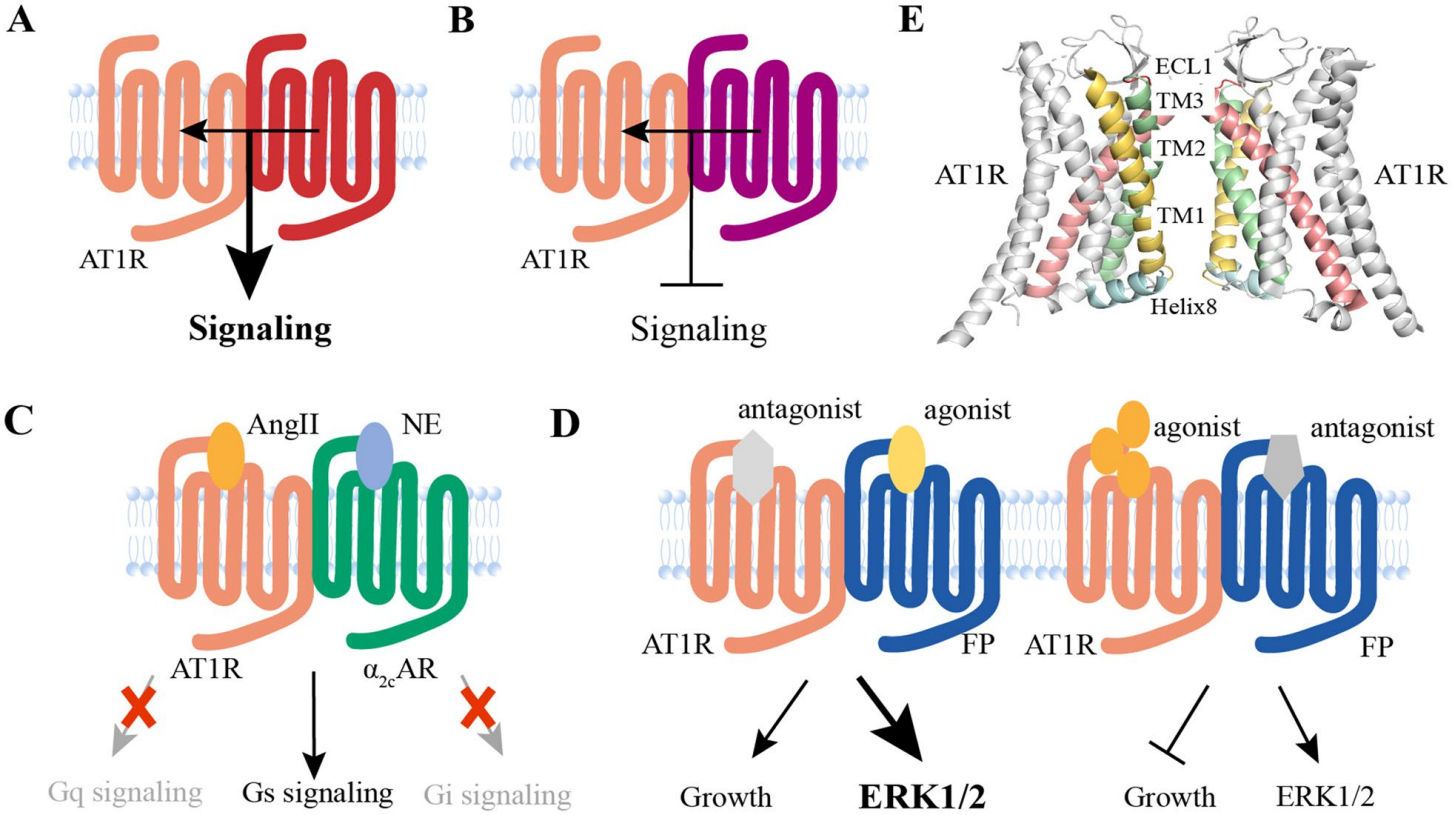
Allostery of AT1R dimers. **A** The heterodimers formed by AT1R with other 7TM receptors could enhance or **B** decrease signaling capabilities. **C** New signaling of AT1R/α_2C_AR heterodimer. **D** Asymmetry of AT1R/FP heterodimer. **E** The interface between the homodimer of AT1R (PDB ID: 6do1) is constituted by ECL1, TM1, TM2, TM3, and Helix 8.

Some receptors that form heterodimers with AT1R can allosterically enhance the potency of AT1R signaling (Fig. 4A). The first receptor to be found to heterodimerize with AT1R was the bradykinin B2 receptor (B2R). The formation of AT1R-B2R enhanced the effect of AngII and triggered the symptoms of preeclampsia in pregnant mice [81, 82]. In addition, heterodimer formation by AT1R and alpha 1D adrenoceptors was barely detected in healthy pregnant rats, but was abundantly found in preeclamptic rats [83]. AngII and thrombin are two key regulators of vascular homeostasis. It has been found that AT1R can interact with the prothrombin receptor (PAR1). Simultaneous activation of the two receptors produced synergistic effects, suggesting positive allosteric interactions. AT1R-PAR1 can be a therapeutic target for coagulation disorder in patients with essential hypertension [84]. Using the appropriate technology and developing drugs that target the reinforcing effect of dimers can greatly improve clinical efficacy and reduce side effects.

Some receptors form heterodimers with AT1R and allosterically antagonize receptor function (Fig. 4B). Angiotensin II type 2 receptor (AT2R) inhibits AngII-induced signaling and antagonizes AT1R in terms of vascular tone and proliferative migration [85, 86]. Ang [1–7] exerts vasodilatory and anti-proliferative effects by binding to MAS receptors encoded by mas proto-oncogenes. MAS receptors inhibit the fuction of AngII by forming heterodimers with AT1R [87]. As an endogenous negative regulator of RAAS, the Apelin-angiotensin receptor-like (APJ) form a heterodimer with AT1R and exert a negative allosteric effect on AngII signaling [88].

Cross-antagonism is a specific form of dimeric allosteric regulation. Single receptor antagonists effectively inhibit both downstream signaling and receptor trafficking. This effect does not interfere with the binding efficacy of the ligand to the receptor, but diminishes the efficacy of one monomer by binding to the other [89]. A dimer was found between AT1R and dopamine D2 receptor (D2R) in the striatum, and antagonists of AT1R inhibited D2R-mediated signaling [90]. Similarly, in the striatum, the combined use of two receptor antagonists inhibited the effects produced by the heterodimerization of adenosine A2A receptor (A2AR) and AT1R. These findings can be helpful for the treatment of tardive dyskinesia (TD) [91]. However, AT1R antagonist losartan enhanced the interaction between AT1R and dopamine D1 receptor (D1R). It might exert anti-hypertensive effects by allosteric enhancement of D1R signaling [92]. After clarifying the allosteric antagonistic function of the dimer, certain drugs may be clinically re-purposed for their unexpected therapeutic effects.

New signaling pathways different from those of monomers or asymmetric signals may form for dimers. In most cases, signaling regulation of dimerization refers to enhanced or diminished activation of the G protein originally coupled to each receptor, but it is possible to form new G protein couplings. A study showed that α2C-adrenergic receptor (α_2C_AR) with NE as its ligand and AT1R with AngII as its ligand could form a heterodimer. However, the concurrent presence of both agonists promoted a new conformation of the dimer, triggering a new form of Gs/cAMP/PKA signaling. It can be a potential new pharmacological target for the treatment of arterial hypertension (HT) and heart failure (HF) [93] (Fig. 4C). AT1R and prostaglandin receptor (FP) formed a heterodimeric complex in HEK293 and vascular smooth muscle cells, forming a new allosteric signaling. It displayed the symmetric and asymmetric signaling behavior depending on how each of the two receptors was affected by agonists or antagonists. Symmetric signaling refers to the fact that one antagonist attenuates the agonistic effect induced by the agonist of another receptor, and one agonist increases the agonistic effect induced by the agonist of another receptor. Asymmetric signaling indicated that antagonists of AT1R greatly enhanced FP-dependent ERK1/2 signaling but had no effect on prostaglandin F2α (PGF2α)-induced cell growth. In addition, antagonists of FP increased the affinity of AngII to AT1R, but inhibited AngII-induced cell growth, and MAPK signaling remained unchanged [94] (Fig. 4D). The emergence of new pathways means the emergence of new therapeutic targets.

## The future of drug design based on AT1R: finding the allosteric sites

Different binding sites of allosteric modulators can lead to different conformational changes of receptor; therefore, the pharmacological effects will be different. A key step in the discovery of new allosteric modulators is the identification of efficient allosteric sites. Due to the lack of visibility of the crystal structure, it is difficult to identify the allosteric sites that are hidden during conformational changes [95]. The discovery of allosteric pockets at protein-protein interfaces is also challenging. The characterization and identification of potential allosteric sites have been considerably improved by rapid progress in kinetic studies and bioinformatics [13, 14].

### Finding the allosteric pockets of AT1R during conformational change

Structural recognition of AT1R facilitates the study of allosteric pockets and the design of allosteric modulators. The transition of receptors from inactivated to an activated state exposes several hidden sites. MD simulations are powerful tools for finding these hidden sites [95]. Two allosteric pockets of AT1R have been identified. Firstly, ECL2 of AT1R is a good design target for allosteric modulators due to its high flexibility and variability [96, 97]. A significant conformational change in ECL2 was discovered from the inactivated to the activated state through MD simulations. Researchers also identified druggable allosteric pockets surrounding the AT1R-AAs epitope. Through high-throughput virtual screening, researchers identified DCP1, a small molecule negative conformational modulator targeting the allosteric pocket, providing a model for the search of other GPCR-AAs inhibitors to intervene in GPCR-AAs-related diseases [98] (Fig. 2B–D). Secondly, the Markov state model (MSM) revealed a mysterious allosteric pocket during AT1R activation and uncovered the kinetic nature of the transition between conformational states. P6 (F^1.48^, L^1.52^, I^1.57^ N^7.49^, P^7.50^, F^7.55^, K^8.49^, F^8.50^, K^8.51^, Y^8.53^, F^8.54^) is a hidden allosteric pocket that transiently exists during dynamic conformational changes. Hidden between TM7 and H8, P6 was only observed during the upward movement of H8. Mutation of this hidden allosteric site can allosterically impair the downstream G protein and β-arrestin signals, helping the design of AT1R allosteric modulators [99] (Fig. 2E). However, these allosteric pockets still require extensive in vivo experiments for validation.

### Finding the allosteric pockets based on the interaction interface of AT1R dimers

In order to successfully design and screen therapeutic agents targeting specific dimers, it is critical to elucidate structural details, such as dimer interaction interfaces and residues [59]. The dimer interaction interface propagates energy perturbations at certain sites of the receptor to neighboring receptors, mediating the synergistic effects of receptors [100]. Allosteric agonists/inhibitors modulate the activation of receptors by acting on the allosteric pocket at the dimer interaction interface [101]. Currently, there is no report of allosteric modulators based on AT1R dimers. Therefore, it is important to identify the interface of specific interactions. Due to the complexity of heterodimers, there is no crystal structure of AT1R heterodimers for interaction interface studies. The interaction interfaces of AT1R homodimer have been well studied. TM4 was central to the study of AT1R homodimers, but mutations in one face of TM4 or both faces of TM4 were not sufficient to completely disrupt homologous AT1R interactions. It has been proposed that at least two interaction interfaces are essential for AT1R homodimer: TM4,5 and TM6,7 [102]. With the help of MD simulations, the four most reasonable mutual interfaces have been proposed: symmetric TM1,2,8, TM5, TM4, and TM4,5 [103]. The only report on AT1R homodimer structure (PDB ID: 6do1) indicates that the interface between individual protomers consists of hydrophobic and aromatic amino acid side chain contacts at ECL1, TM1, TM2, TM3, and H8 [104] (Fig. 4E). Interestingly, AT1R homodimers mediated β-arrestin signaling, but not Gq signaling [105]. The formation of homodimer interfaces may allosterically modulate the downstream signal. Some researchers proposed a “rolling dimer” interface model, in which several conformations of the dimer coexist and interconvert [106], providing a new concept for dimer interaction interface in physiological conditions.

## Conclusion

Classical and non-classical allostery are critical pathways depending on GPCR activation, which creates unlimited possibilities of cellular and biological functions. Distinctively, biased agonists and dimers of AT1R both exert unique functions through allosteric modulation, alter ligand recognition patterns, and change receptor core cascade and downstream signal transduction. In particular, the discovery of allosteric regulators improved our understanding of the signaling and functional diversity of AT1R in physiological and pathological conditions. The discovery of allosteric sites provides new opportunities for developing allosteric drugs. Despite great progress in the structural identification of AT1R, the structures of non-antibody allosteric activators in complex with AT1R, especially the extracellular loops, are yet unknown and do not meet the current needs for allosteric drug development. In addition, the bidirectional communication in the allosteric regulation of AT1R needs more studies. Currently, the development of allosteric modulators of GPCR is in progress. Computational biology, bioinformatics, and experimental methods can accelerate the development of allosteric modulators targeting AT1R.

## Data Availability

All data are included in the manuscript.

## Abbreviations

AT1R: Angiotensin II type 1 receptor
ARB: AT1R blocker
GPCRs: G protein-coupled receptors
TM: Transmembrane
ECL1-3 and ICL1-3: Extra- and intracellular loops
H8: Helix 8
PAM: Positive allosteric modulators
NAM: Negative allosteric modulators
NAL: Neutral allosteric ligands
ago-PAM: Ago-allosteric modulator
BAM: Biased allosteric modulators
AT1R-AAs: AT1R autoantibodies
MD: Molecular dynamics
COMP: Cartilage oligomeric matrix protein
TSHR: Thyroid-stimulating hormone receptor
CAMs: Constitutively active mutants
FP: Prostaglandin receptor
α2CAR: α2C-adrenergic receptor
